# Quality characteristics and consumer perception of non-alcoholic beers in the context of responsible alcohol consumption

**DOI:** 10.1038/s41598-025-89833-0

**Published:** 2025-02-28

**Authors:** Anna Gliszczyńska-Świgło, Inga Klimczak, Dorota Klensporf-Pawlik, Iga Rybicka

**Affiliations:** Institute of Quality Science, Poznań University of Economics and Business, al. Niepodległości 10, Poznań, 61-875 Poland

**Keywords:** Beer quality, Non-alcoholic beer, Consumer perception, Responsible alcohol consumption, Sustainable development goals, Environmental social sciences, Chemistry

## Abstract

Consumers are becoming more aware of the benefits of reducing alcohol consumption, which has increased the popularity of non-alcoholic beer (NAB). However, the brewing industry still faces many difficulties in producing NAB with similar physicochemical and sensory characteristics to regular beers. To mitigate these challenges, a multidisciplinary study was conducted to: (1) determine selected quality characteristics of various NABs, (2) compare them with literature data for regular beers, and (3) find out how Polish consumers, especially young people, perceive NAB and what motivates them in their purchasing decisions. Physicochemical quality characteristics of NABs, namely pH (4.3–4.7), bitterness (IBU = 12–77), colour (EBC = 6.4–19.6), total soluble solids (2.8–8.3 °Bx), and vitamin B_2_ content (0.11–0.30 mg/L) were similar to alcoholic beers. Total phenolics (150–321 mg/L), Ca (13–45 mg/L), Mg (40–94 mg/L), K (192–490 mg/L), Na (0.5–57 mg/L), and P (158–467 mg/L) contents in NABs were slightly lower than in alcoholic beers, but this assortment is a healthier source of these nutrients due to the lack of alcohol-related harm. According to the consumer study, taste and availability in shops were found to be crucial factors influencing NAB purchasing decisions.

**Supplementary Information:**

The online version contains supplementary material available at 10.1038/s41598-025-89833-0.

## Introduction

It is estimated that the use of alcohol causes about 3 million deaths every year and is the leading risk factor for public health − 5.3% of all deaths with a significant proportion of the young^1^. Therefore, in recent years, various stakeholders, including producers of alcoholic beverages, have made commitments and actions to support responsible alcohol consumption. These are, for example, “EU Alcohol Strategy” adopted in 2006 by the European Commission^2^ or the Sustainable Development Goals introduced by the United Nations in 2015^3^. Producers sustainability strategies also often refer to the World Health Organization “Global Strategy to Reduce the Harmful Use of Alcohol” or to “Global alcohol action plan 2022–2030” established to effectively implement the global strategy to reduce the use of alcohol as a public health priority^1,4^. Their activities and market campaigns are also aligned with the United Nations Sustainable Development Goals such as Goal 3 – Good health and well-being, Goal 12 – Responsible consumption and production, and Goal 17 – Partnership for the goal^5,6^. All of the above aim to improve the health of individuals, families and entire local, national and global communities, with a considerable reduction in the morbidity, mortality and social consequences caused by the harmful use of alcohol.

The average consumption of alcohol by people aged 15 and over in European countries ranged from 6.3 to 12.2 L per capita in 2022^7^. In 2019, about 8% of the EU adult population consumed alcohol daily, 29% weekly, 23% monthly and 26% never consumed or had not consumed any in the last 12 months. Responsible drinking is understood as a model of occasional, moderate consumption of alcoholic beverages by healthy adults and drinking non-alcoholic beverages among vulnerable populations. Moderate alcohol consumption by healthy individuals, defined as no more than one drink for women and two drinks for men per day, is associated with decreased mortality resulting from the reduced risks for cardiovascular disease and type 2 diabetes^8,9^. For example, according to the Dietary Guidelines for Americans, one alcohol drink-equivalent contains 14 g (0.6 fluid ounces; fl oz) of pure alcohol, which is equivalent to 12 fl oz (about 350 mL) of beer with 5% ABV, 5 fl oz (about 150 mL) of wine (12% ABV), or 1.5 fl oz (about 44 mL) of 80 proof distilled spirits (40% ABV)^10^. Simultaneously, frequent heavy drinking leads to physical and mental diseases such as heart disease, liver disorders, obesity, various types of cancer, and dementia. As a part of the commitment to responsible consumption of alcoholic beverages, producers have expanded their product portfolio by introducing alcohol-reduced or alcohol-free products (e.g^5,11^).

Beer is a refreshing product appreciated by consumers around the world for its taste and aroma. It is the most consumed alcoholic beverage in the world and the third most popular beverage after water and tea^12,13^. The global consumption of beer was approximately 192 million kilolitres in 2022 (2.9% more than in the previous year), with Czech Republic, Austria, Poland, Republic of Ireland, Lithuania, Spain, Germany, Estonia, Romania, and Namibia being the top 10 countries with the highest per capita consumption^14^. Growing consumer concerns about health issues and the growing popularity of non-alcoholic beverages have prompted breweries to expand their assortment of non-alcoholic beers (NAB)^12,15^. Thus, in recent years, a significant increase in the consumption of NAB has been observed around the world. NAB typically refers to products with no or low alcohol content (≤ 0.5% ABV), but its definition may vary by country^16^. The worldwide market of NAB has increased from 3,530 million litres in 2008 to 6,504 million litres in 2022 with the highest share of two companies: Heineken NV (18% of the total volume) and Anheuser-Busch InBev NV, producer of e.g., Budweiser and Corona brands (16%)^17^. In 2023, the global NAB market generated revenue of about 35 billion U.S. dollars and is expected to grow up to 46 billion dollars in 2027^18^. The great popularity of NABs results from consumers’ preferences, as these products allow them to enjoy the taste and aroma of beer without giving up their daily habits, such as driving or physical activity^15^. Regular and NAB are also a good source of some water-soluble vitamins, especially vitamin B_2_, minerals and antioxidant phenolic compounds. From a nutritional point of view, the most important are macroelements, namely calcium (Ca), potassium (K), magnesium (Mg), phosphorous (P) and sodium (Na), as well as other compounds such as phenolic compounds which influence the taste of beer and its total antioxidant capacity. Therefore, the consumption of NAB or moderate consumption of alcoholic beer can be an important source of minerals and some B-group vitamins during exercise or hot days, and can also be a source of health-promoting phenolic compounds^19,20^. All these compounds come from ingredients used for brewing such as water, malt grains, hops, yeasts, spices, or fruits (if used) and are related to processes involved in beer production.

In order to comment on the possible impact of NABs on the quality of life, we decided to evaluate selected quality characteristics of these products and their potential role in limiting alcohol consumption understood as a prospective shift from the consumption of traditional beer to NAB. To the best of our knowledge, this is a new approach that supports the global strategy to reduce alcohol consumption and fits into the Sustainable Development Goals. What is more, the British studies revealed that buying and consuming NAB is much more likely to occur in younger age groups^21^. Other data also showed that an increasing percentage of young Germans and Poles are giving up alcohol consumption^22^. Therefore, on the one hand, the aim of the study was to assess selected quality characteristics of NABs, namely: pH, total soluble solids (TSS), colour, bitterness, the total phenolic content (TPC), macroelements (Ca, K, Mg, Na, P), and vitamin B_2_ content. Selected IPA, brand lager, and wheat beers were evaluated and their quality characteristics were compared with literature data concerning their alcoholic counterparts. On the other hand, the consumer perspective was described in relation to the perception and consumption of NAB by young consumers. All of the above natural and social attributes have direct implications and impact on the quality and well-being of beer consumers in their everyday lives. They can also be important from the perspective of beer producers whose marketing campaigns target NABs and support the idea of responsible alcohol consumption.

## Results

### Quality characteristics of NABs

In the present study, selected quality characteristics: bitterness, pH, colour, TSS, TPC, as well as the concentration of vitamin B_2_ and macroelements in 15 NABs of three types (IPA, brand lager, and wheat beers) were determined (Tables 1 and 2) and compared to existing data for regular (with alcohol) beers.

**Table 1:** Quality characteristics of non-alcoholic beers: pH, bitterness, colour, total soluble solids (TSS), total phenolic content (TPC) and vitamin B_2_.

Beer		pH	Bitterness	Colour	TSS	TPC	Vitamin B_2_
		[-]	IBU	EBC units	^o^Bx	mg GAE/L	mg/L
IPA	1	4.42 ± 0.004	23.2 ± 1.0	6.97 ± 0.01	5.8 ± 0.0	192 ± 6	0.167 ± 0.0007
	2	4.72 ± 0.001	50.4 ± 1.0	7.80 ± 0.00	5.7 ± 0.1	191 ± 11	0.180 ± 0.0003
	3	4.67 ± 0.006	56.0 ± 1.0	8.89 ± 0.01	6.2 ± 0.0	189 ± 3	0.156 ± 0.0008
	4	4.62 ± 0.001	76.5 ± 0.9	8.11 ± 0.01	6.7 ± 0.1	321 ± 12	0.169 ± 0.0001
	5	4.36 ± 0.003	30.4 ± 0.9	9.03 ± 0.03	5.2 ± 0.0	150 ± 5	0.114 ± 0.0004
	Average	4.56 ± 0.16^a^	47.3 ± 21.2^a^	8.02 ± 0.97^a^	5.9 ± 0.6^a, b^	209 ± 65^a^	0.157 ± 0.025^a^
Brand lager	6	4.57 ± 0.001	38.5 ± 0.7	10.32 ± 0.01	6.5 ± 0.0	225 ± 8	0.172 ± 0.0010
	7	4.33 ± 0.001	34.4 ± 0.7	10.23 ± 0.04	4.8 ± 0.1	169 ± 8	0.166 ± 0.0004
	8	4.59 ± 0.002	54.4 ± 0.7	9.46 ± 0.01	5.7 ± 0.0	191 ± 3	0.163 ± 0.0002
	9	4.35 ± 0.000	24.3 ± 0.3	12.07 ± 0.03	3.2 ± 0.0	191 ± 11	0.302 ± 0.0002
	10	4.33 ± 0.001	25.7 ± 0.6	10.56 ± 0.01	2.8 ± 0.0	176 ± 4	0.109 ± 0.0002
	Average	4.43 ± 0.13^a^	35.5 ± 12.2^a, b^	10.53 ± 0.95^a^	4.6 ± 1.6^b^	190 ± 21^a^	0.182 ± 0.072^a^
Wheat	11	4.31 ± 0.000	28.7 ± 1.2	15.25 ± 0.03	7.2 ± 0.1	236 ± 6	0.153 ± 0.0003
	12	4.39 ± 0.001	29.9 ± 0.6	10.43 ± 0.03	7.3 ± 0.0	205 ± 5	0.112 ± 0.0005
	13	4.29 ± 0.000	33.0 ± 0.5	6.35 ± 0.00	8.3 ± 0.1	201 ± 4	0.131 ± 0.0003
	14	4.60 ± 0.002	22.5 ± 0.3	19.63 ± 0.04	6.9 ± 0.0	300 ± 12	0.169 ± 0.0004
	15	4.38 ± 0.006	12.3 ± 0.2	7.05 ± 0.02	5.4 ± 0.0	182 ± 5	0.155 ± 0.0004
	Average	4.40 ± 0.12^a^	25.3 ± 8.2^b^	11.74 ± 5.64^a^	7.0 ± 1.0^a^	225 ± 46^a^	0.144 ± 0.022^a^

a-b: significant differences (*p* < 0.05) between average values for beer types are indicated in the columns by different letters.

**Table 2:** Quality characteristics of non-alcoholic beers: minerals.

Beer		Ca	Mg	K	Na	P
		mg/L	mg/L	mg/L	mg/L	mg/L
IPA	1	30.3 ± 0.6	63.4 ± 0.7	295 ± 3	13.0 ± 1.8	192 ± 3
	2	36.6 ± 0.6	69.0 ± 0.8	353 ± 7	57.1 ± 1.5	217 ± 10
	3	38.3 ± 0.8	76.5 ± 1.1	420 ± 9	56.5 ± 1.2	176 ± 3
	4	37.0 ± 0.5	71.8 ± 1.0	430 ± 2	36.5 ± 1.5	263 ± 8
	5	37.0 ± 0.2	51.8 ± 0.5	258 ± 2	4.6 ± 0.5	252 ± 9
	Average	35.8 ± 3.2^a^	66.5 ± 9.5^a, b^	351 ± 75^a^	33.5 ± 24.2^a^	220 ± 37^a^
Brand lager	6	38.0 ± 0.6	58.3 ± 1.3	303 ± 6	7.6 ± 0.5	466 ± 14
	7	38.6 ± 0.8	45.0 ± 0.7	192 ± 3	2.5 ± 0.1	186 ± 6
	8	38.0 ± 0.1	52.3 ± 0.2	267 ± 1	6.7 ± 0.1	419 ± 11
	9	24.2 ± 0.1	51.0 ± 0.8	308 ± 5	8.7 ± 0.05	161 ± 8
	10	21.9 ± 0.1	45.9 ± 1.1	275 ± 7	8.5 ± 0.5	158 ± 5
	Average	32.1 ± 8.3^a^	50.5 ± 5.4^b^	269 ± 46^a^	6.8 ± 2.5^b^	278 ± 151^a^
Wheat	11	13.4 ± 0.4	69.7 ± 0.8	341 ± 8	12.2 ± 0.8	330 ± 11
	12	45.2 ± 0.9	67.3 ± 1.1	341 ± 3	0.5 ± 0.03	255 ± 8
	13	43.4 ± 0.8	79.8 ± 2.0	321 ± 5	36.2 ± 0.6	341 ± 5
	14	38.5 ± 0.2	93.5 ± 0.8	490 ± 3	8.4 ± 0.4	467 ± 12
	15	30.2 ± 0.6	40.2 ± 0.3	201 ± 1	2.6 ± 0.02	214 ± 7
	Average	34.1 ± 14.0^a^	70.1 ± 19.6^a^	339 ± 103^a^	12.0 ± 14.3^a, b^	321 ± 97^a^

a-b: significant differences (*p* < 0.05) between average values for beer types are indicated in the columns by different letters.

The pH values of NABs in the present study ranged from 4.29 to 4.72. Their colour, in EBC units, was 6.4–19.6. No statistically significant differences were noticed between IPA, brand lager, and wheat beers for these parameters (*p* > 0.05). The bitterness of brand lager NABs was 24.3–54.4 IBU. For IPA beers, the IBU ranged from 23.2 to 76.5, and for wheat NABs – from 12.3 to 33.0 IBU. The TSS content in beers tested ranged from 2.8 to 8.3 °Brix. On average, the highest TSS content was in wheat (7.0 °Brix), followed by IPA (5.9 °Brix) and brand lager NABs (4.6 °Brix). The concentration of phenolic compounds in IPA NABs was 150–321 mg/L, in brand lagers NABs – 169–225 mg/L, and 182–300 mg/L in wheat NABs. The concentration of vitamin B_2_ was 0.11–0.18 mg/L, 0.11–0.30 mg/L, and 0.11–0.17 mg/L in IPA, brand lagers, and wheat NABs, respectively. No significant differences between the average concentration of phenolic compounds and vitamin B_2_ were noticed for three groups of beers (*p* > 0.05) (Table 1).

The content of minerals in NABs under the study is presented in Table 2. No significant differences were noticed (*p* > 0.05) for Ca (13–45 mg/L), K (192–490 mg/L), and P (158–467 mg/L) between IPA, brand lager, and wheat types of beer. The average concentration of Mg was the highest in wheat beers (40–94 mg/L), followed by IPA (52–77 mg/L) and brand lager beers (46–58 mg/L), but the differences between wheat and IPA beers, as well as between IPA and brand lager beers were not statistically significant (*p* > 0.05). The lowest concentration of Na was found in brand lager beers (2.5-9 mg/L) and wheat beers (0.5–36 mg/L), followed by IPA (4.6–57 mg/L).

**Fig. 1:**
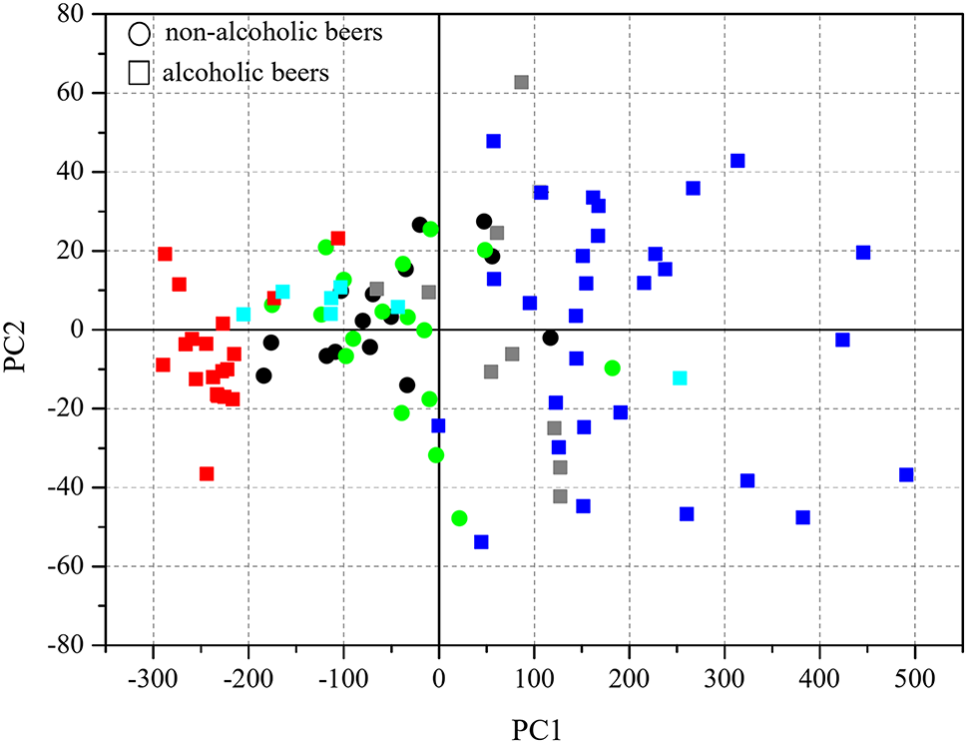
Principal Component Analysis of Ca, Mg, K, Na, and P in non-alcoholic and regular (alcoholic) beers. Black circle − non-alcoholic beers of the present study; green circle − non-alcoholic beers analysed by Alcázar et al.^44^; grey square − alcoholic beers analysed by Alcázar et al.^44^; blue square − alcoholic beers analysed by Zambrzycka-Szelewa et al.^20^; red square − alcoholic beers analysed by Voica et al.^48^; cyan square − alcoholic beers reported in USDA^33^.

Figure 1 shows the results of Principal Component Analysis (PCA) performed for the concentration of Ca, Mg, K, Na and P in the tested NABs compared to literature data for non-alcoholic and regular beers from Poland, Romania, and Italy. PCA was used to visualize macroelement concentration data for the beers studied (Table 2) and literature data for other non-alcoholic and alcoholic beers. Visualization of multivariate experimental and literature data allows to see the main similarities and differences in macroelement content between non-alcoholic and alcoholic beers (the numerical literature data are discussed in the Discussion section). As far as we know, such a graphical comparison of non-alcoholic and regular beers is not available in the literature. The first and the second principal components (PC1 and PC2) explained 96% and 2% of the total data variance, respectively. Generally, the concentration of macroelements in NABs is slightly lower than in regular beers, but they are a healthier source of macroelements than alcoholic beers due to the lack of alcohol-related harm. Just half a litre of NAB under the study can deliver up to 12–13% of the recommended daily intake for Mg and K, and 33% for P with less than 1.5% of recommended daily intake of (nutritionally undesirable) Na.

### Consumer study

**Fig. 2:**
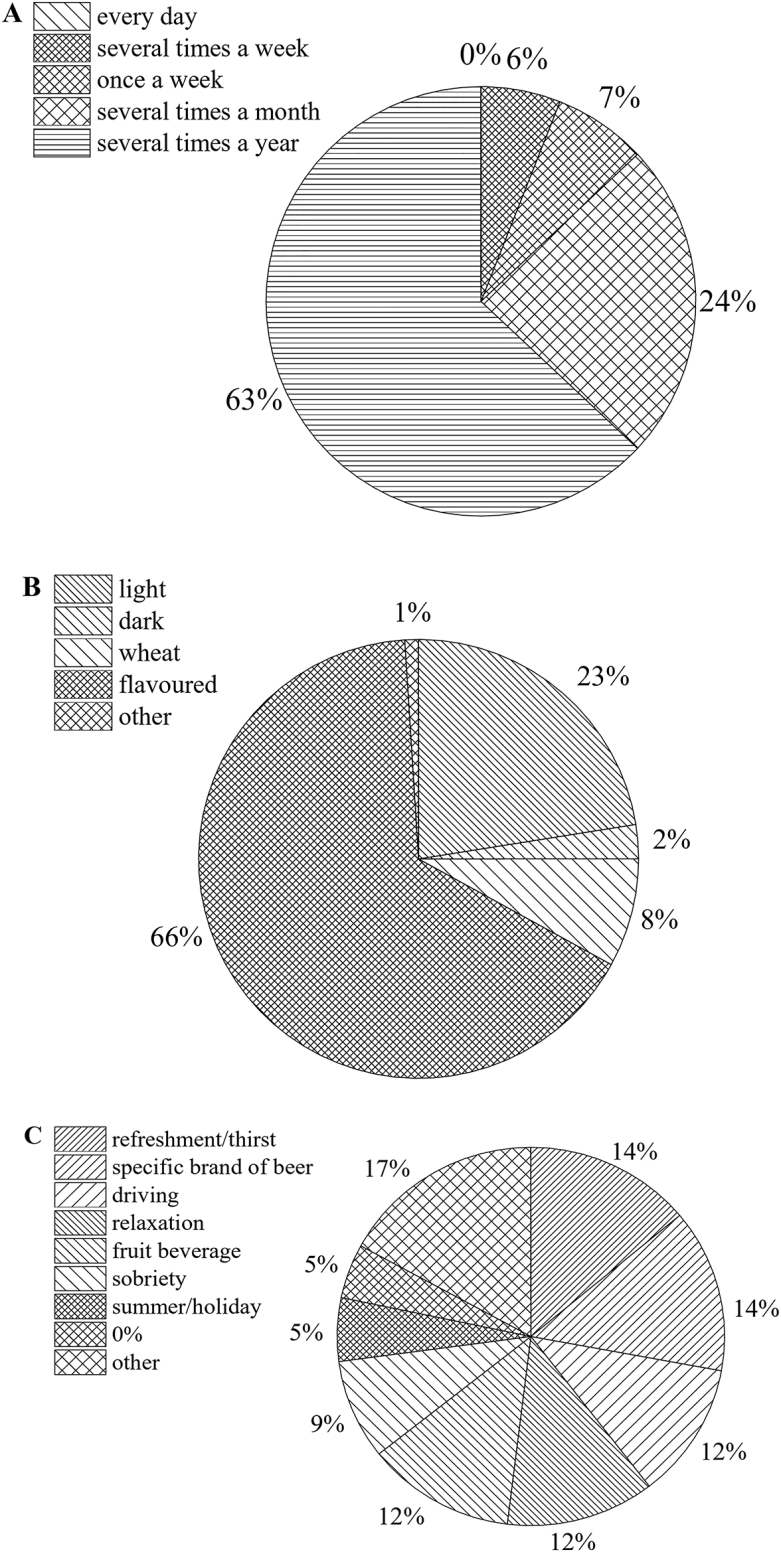
Frequency of purchase of non-alcoholic beers by consumers (**A**), type of non-alcoholic beer preferred by consumers (**B**) and the words associated with non-alcoholic beer (**C**).

The participants of the present study were mostly young people, approximately 45% of respondents were aged 18–25 and 40% were aged 26–35. About 88% of them lived in the city and about 50% perceived their income as high or very high (Table 3). No one declared that they drink NAB every day, about 63% drink NAB several times a year, and as many as 24% drink it several times a month (Fig. 2A). The respondents were also asked what type of NAB they consume most often. Light beer (in terms of colour) was consumed by nearly 23% of questioned persons, dark beer by 2% and wheat beer by 8%. Among the 132 (66% of participants) who declared drinking flavoured beer, 64% were women and 36% were men (Fig. 2B). The next question was about the word(s) they associate with NAB (Fig. 2C). Of those who answered this question, about 14% indicated refreshment/thirst or a specific beer brand. About 12% of persons associated NAB with driving or relaxation, or a fruit beverage, and about 9% with sobriety. For 5%, NAB was associated with summer/holidays or 0% (alcohol). The rest of the respondents (17%) provided answers that could not be combined into groups. Moreover, about 5% of participants referred to NAB as a product with no alcohol, whereas it can contain up to 0.5% by volume depending on the definition adopted by a country.

**Table 3:** Socio-demographic characteristic of survey participants.

Feature	Category	Frequency	%
Gender	Female	132	63.2
	Male	77	36.8
Age (Years)	Below 18	1	0.5
	18–25	94	45.0
	26–35	84	40.2
	36–45	7	3.3
	46–55	12	5.7
	Over 55	11	5.3
Place of residence	Village	25	12.0
	City below 50,000 inhabitants	16	7.7
	City 50,000-150,000 inhabitants	55	26.3
	City 150,000-500,000 inhabitants	40	19.1
	City over 500,000 inhabitants	73	34.9
Education	Vocational	4	1.9
	Primary	3	1.4
	Secondary	46	22.0
	Higher engineer/bachelor	83	39.7
	Higher (master degree)	73	34.9
Occupation	Student (high school)	3	1.4
	Student (first degree)	22	10.5
	Student (second degree)	56	26.8
	Unemployed	6	2.9
	Housekeeping	3	1.4
	White collar	98	46.9
	Blue collar	18	8.6
	Retired	3	1.4
Income	No income	9	4.3
	Not sufficient	3	1.4
	Can afford only basic or selected thing	90	43.1
	Can afford anything	37	17.7
	Can afford anything and save	70	33.5
Non-alcoholic beer consumption	Yes	200	95.7
	No	9	4.3

**Fig. 3:**
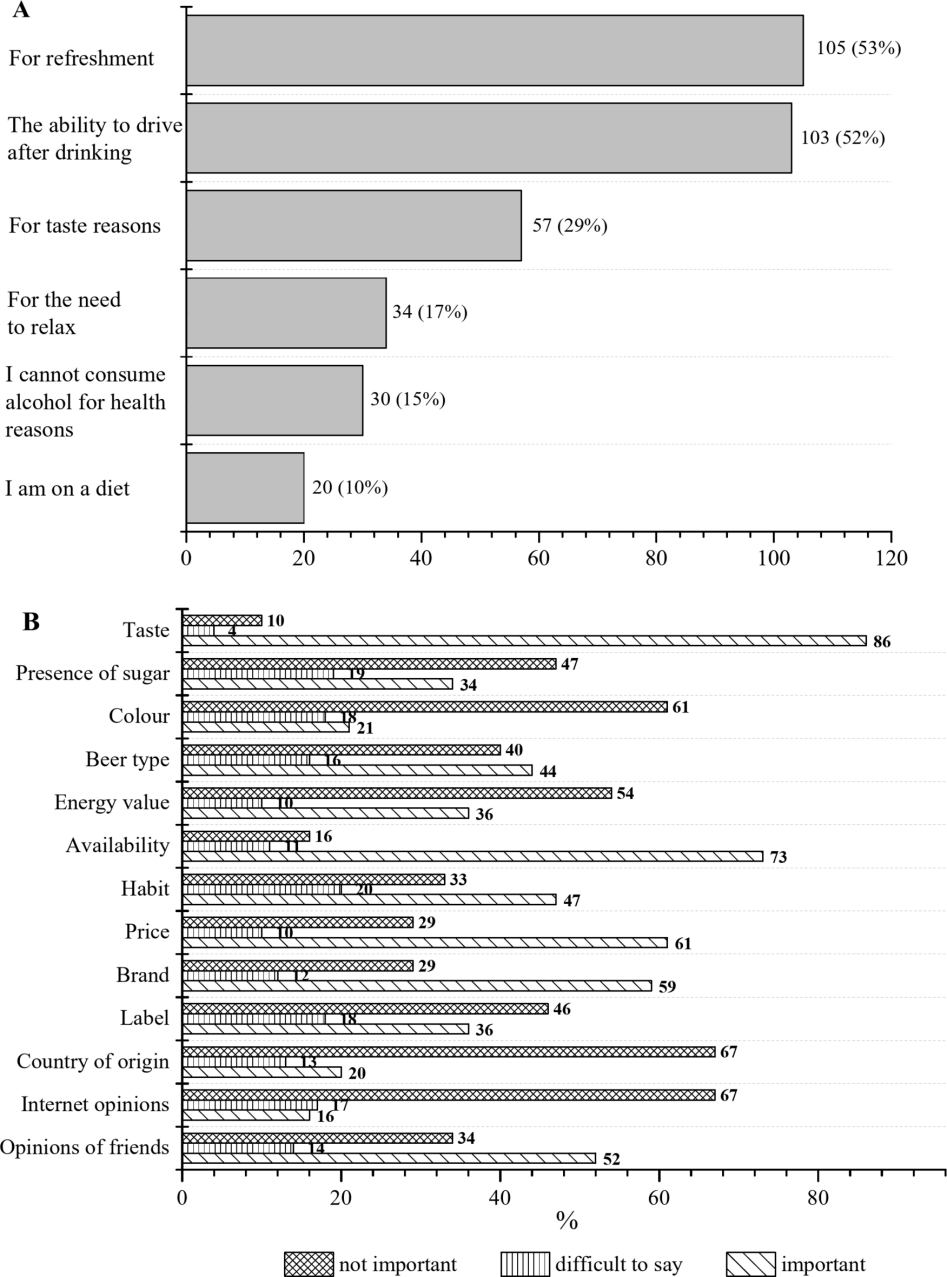
Consumer motivations behind the choice of non-alcoholic beer (**A**) and percentage of respondents according to factors influencing the decision to purchase non-alcoholic beers (**B**).

The respondents of the present study were also asked about their motivation to buy NAB (Fig. 3A) and the factors influencing their purchasing decision (Fig. 3B). These factors included: beer type, brand, country of origin, label, taste, colour, presence of sugar, energy value, availability, price, habit, opinion of friends, and opinions found on the Internet. A chi-square test showed that there were no significant differences between the indications of men and women, except for the presence of sugar. This factor was more important for women than men (*p* < 0.05). As the motivation for purchasing NABs, the questioned most often indicated the possibility of driving after drinking (about 52%) and the refreshment offered by NABs (about 53%). Nearly 29% of persons declared that they reach for NAB for its taste, 17% for the need to relax with a beer and less than 15% for health and dietary reasons (Fig. 3A). Important factors influencing their decision to purchase NABs were: taste (86%), availability (73%), price (61%), brand (59%), friends’ opinion (52%), and habit (47%) (Fig. 3B). Unimportant for the consumers surveyed in the present study were: opinions found on the Internet (67%), country of origin (67%), and colour (61%). The energy value of beer and the presence of sugar were important for 36% and 34% of respondents, respectively. As many as 46% considered product label unimportant, while 36% considered it vital indicating a wide discrepancy in the importance attached to the visual appearance of the product.

## Discussion

The popularity of beer is due to its pleasant sensory characteristics and favourable nutritional properties, as well as its lower price compared to other types of alcoholic beverages^12,13^. However, growing consumer awareness of the negative effects of alcohol consumption was one of the factors that influenced the growth of the NAB market. The increasing consumption of NABs has resulted in research and innovations in their production to provide products with the lowest possible alcohol content that are very similar to their alcoholic counterparts^23^.

The results of the present study showed that the pH (4.29–4.72) and colour (6.4–19.6 EBC) of the beers tested were similar to regular beers (pH of 4.0-4.8 and EBC below 26 for light beers, respectively). Bitterness is an essential quality parameter of beer. It depends primarily on the amount of iso-alpha-acids originating from the hops. The IBU values for beer may range from 0 to 100. Generally, lager beers have IBUs ranging from 15 to 50, but e.g. American lagers tend to be lower on the IBU scale, usually in the 8–18 range^24^. The bitterness of brand lager NABs of the present study (24.3–54.4 IBU, Table 1) was generally higher than that reported by Ramsey et al.^25^ and Lafontaine et al.^26^ for lager NABs (5.3–25.3 and 8.4–33.5 IBU, respectively), but similar to traditional beers. Three of five IPA NABs had IBUs above 50, which corresponds to the typical bitterness of alcoholic IPA beers (50–70 IBU)^24^. In the study of Lafontaine et al.^26^, IPA NABs had bitterness in the range of 12.4–56.9 IBU. Wheat ales are lower in bitterness than their corresponding barley counterparts resulting in relatively low, 8–35, IBU^24^. The IBU values for wheat NABs of the present study (12.3 to 33.0) were similar to the results obtained by Lafontaine et al.^26^ for wheat NABs (10.8–16.6 IBU). The TSS content of Brazilian craft (alcoholic) beers (5.1–11.0 °Brix)^27^ was generally comparable to the TSS of the NABs from the present study (2.8–8.3 °Brix). In the study of Blackmore et al.^28^, beers labelled as 0.0% ABV were expected or perceived as less bitter, having a less full body and were less liked than beers labelled as 4.5% ABV. Beers with a darker colour were expected to be more bitter, but no effect of colour on perceived bitterness was observed. In contrast, Lafontaine et al.^29^ reported that American consumers were less satisfied with NABs perceived as more beer-like in terms of aroma, taste, and mouthfeel (i.e. bitterness). The study of Ramsey et al.^30^ on the influence of ethanol concentration on liking and sensory attributes of lager beer showed that bitterness had a positive influence on the liking of 0% ethanol samples but a negative on the liking of 2.8% and 5% ethanol samples. Ivanova et al.^31^ observed the positive impact of ethanol on beer body intensity at the low bitterness, but not at the high bitterness suggesting that higher bitterness is required in lower-alcohol beers to achieve a similar body intensity response in consumers. On the other hand, higher bitterness had a negative effect on overall liking. Both, Ramsey et al.^30^ and Ivanova et al.^31^, observed individual differences within a population of consumers tested. They could divide consumers into different groups showing the liking or disliking of beer containing different ethanol levels, including a cluster that liked low/no alcohol beer products similarly to standard beers^30^ or according to the perceived beer body intensity depending on alcohol concentration^31^. Thus, a NAB may be perceived as sensory appealing and similar to or even better than an alcoholic beer. Both studies suggested that at different concentrations of ethanol, different attributes are enhanced or masked, which may affect liking/disliking differently even within the same population of consumers.

The content of vitamin B_2_ in beer varied from 0.11 mg/L to 0.30 mg/L, with riboflavin as the main form of vitamin B_2_. These results are consistent with literature data for traditional beers (0.12–0.33 mg/L)^32,33^, making NAB a good source of this vitamin. Half a litre of NABs tested in this study can provide up to 4–11% of the recommended daily intake of this vitamin. The concentration of riboflavin in beer is not only important from a nutritional point of view. This compound is a photosensitizer in the formation of a sunstruck flavour in beer and may therefore affect negatively the sensory quality of beer^34^.

Beer is a good source of compounds with antioxidant activity such as phenolic compounds. They include phenolic acids, flavonoids, proanthocyanidins, tannins and amino phenolic compounds. The phenolic profile of different types and brands of beers is similar, but they differ in the concentration of individual compounds and the total phenolic content. This is due to the differences between raw materials and beer production technology. About 70–80% of beer phenolics originate from malt, whereas 20–30% come from hops^35,36^. All these compounds influence the sensory attributes of beer, mainly flavour, colour, bitterness, and astringency^37^, as well as the antioxidant capacity of beer. NABs of this study contained from 150 mg GAE/L to 321 mg GAE/L, which is in line with results obtained by Ramsey et al.^25^ for 18 non-alcoholic lagers (43–236 mg GAE/L). TPC in regular barley and wheat beers is various, depending on the type and country of origin, with dark beers usually having a higher TPC than light beers. For example, TPC in beers available on the Polish market may range from 291 to 1266 mg GAE/L^20,38^, while in Serbian or Italian beers – 330–545 and 274–424 mg GAE/L, respectively^39,40^. The lower TPC in NAB can be attributed to the brewing process, where less concentrated wort can be used to limit the fermentation. Dealcoholisation of beer may also contribute to the loss of phenolic compounds^41^. Although TPC tends to be lower in NABs than in alcohol-containing ones, NABs can be considered a good source of phenolic antioxidants due to the possibility of their higher intake from NAB than from regular beers. Moreover, there is a potential to enhance the bioactive compounds, including (poly)phenols, in NAB through innovative brewing approaches in terms of ingredients (e.g. addition of fruits, fruit juices, parts of various plants, and plant extracts), brewing methods, and type of fermentation^42,43^.

The concentrations of minerals in NABs in this study are in line with the results for NABs studied by Alcázar and co-authors^44^: Ca (29–108 mg/L), K (200–555 mg/L), Mg (43–97 mg/L), P (108–297 mg/L), and Na (8–82 mg/L), but lower than literature data for minerals in regular beers: Ca (3-140 mg/L), K (30-1100 mg/L), Mg (20–220 mg/L), P (65–400 mg/L) and Na (1-230 mg/L)^20,45–48^. PCA results (Fig. 1) of Ca, Mg, K, Na, and P in non-alcoholic and regular beers confirmed some differences between these products in terms of macroelements, but, as with phenolic compounds, NAB may be a better source of minerals due to the ability to drink more NAB than its alcoholic counterpart.

To date, numerous studies have been conducted on factors influencing the consumption of no and low alcoholic drinks, including NAB. However, the need to analyse differences in the acceptance and consumption of these products in different geographic regions, religions, and national cultures is still pointed out. Providing a cross-cultural analysis will allow a better understanding of the spread of no and low alcoholic drinks consumption in different regions^15^. To meet these expectations, the quantitative survey was designed to evaluate Polish consumers’ attitudes towards NABs.

The participants of the present study were mainly young people, as the largest groups consuming NABs, for example in Germany, the United Kingdom, and the United States are ‘Millennials’ and ‘Generation X’, who make up 52–81% of NAB drinkers, as reported in a large cross-country survey conducted in 2022^49–51^. About 96% of our respondents declared that they had consumed NAB at least once in their lives, which is more common than in the general population, comprising various age groups^16^. According to the latest report of Kompania Piwowarska (one of the largest beer producers in Poland)^52^, about 25% of NAB consumers drink this beverage several times a month, which is in line with the results of our study (24%, Fig. 2A). From a global perspective, there are various reasons people do not drink alcohol, including religion, health benefits, personal preferences or lifestyle habits such as driving. Moreover, NAB buyers are most often referred to as highly educated and high-income consumers^15^, which is in line with the findings of our study. Anderson et al.^20^ reported that buying and consuming NAB is much more likely to occur in younger age groups, more affluent households and those with higher social grades. Similar results were obtained in this study – about 50% of respondents indicated their income as high or very high. The data obtained within the Statista Consumer Insights Global survey indicated that consumers of NABs are more likely to live in cities and urban areas than the average consumer, and they are predominantly male^49–51^.

The word(s) that Polish consumers most frequently associated with NAB were refreshment/thirst, a specific beer brand, driving or relaxation, a fruit beverage and sobriety. These features were also indicated as important elements in the consumer assessment of NABs conducted by other researchers, e.g^16,28^. The association of NAB with a fruit beverage is not surprising in light of the fact that respondents indicated flavoured beer as the most commonly consumed (Fig. 2B). Interestingly, blue and white colours are globally associated with not flavoured alcohol-free beverages and this unwritten rule is used by the manufacturers of the most popular global brands such as Heineken or Becks^53^.

According to our study, the most important factors influencing the decision to purchase NABs were: taste, availability, price, brand, friends’ opinion, and habit (Fig. 3B). Taste was also the most important for Brazilian craft beer consumers^54^. Availability in shops, as a factor influencing purchase decisions, is in line with the result of a survey presented by SW Research^55^. This survey showed that, on the one hand, producers are offering an increasing choice of good-tasting products and, on the other hand, consumers are looking for greater availability, quality, and a wider choice of NABs and are more willing to buy zero alcohol beverages. This is a noticeable trend − 72% of Poles noticed an increasing choice of high quality NABs in stores^55^.

Unimportant for the consumers surveyed in the present study were: opinions found on the Internet, country of origin, and colour. The energy value of beer and the presence of sugar were important for about 1/3 of respondents. In the study of da Costa Jardim et al.^54^, the calories in regular Brazilian craft beers were relevant for only 13% of respondents. It is worth noting that the colour and appearance of a product are certainly important descriptors of the product and they can influence purchasing decisions, but consumers cannot always judge these factors as the packaging often hides the product. With this, it can be explained why for 79% of respondents the colour of the beer was not important or was neutral when buying a particular product. It is also important regarding the common belief that NAB has a worse taste than regular beer. For example, Staub and co-authors^56^ investigated how different factors influence NAB consumption frequency in the German population. They confirmed that, despite the enormous effort taken in recent years to improve the taste of NABs, most consumers still support this opinion. It is in line with other studies showing that the opinion of a beer may be the result of its labelling as alcohol-free rather than its actual taste^57^.

## Conclusions

Today’s society is aware of the benefits of reducing alcohol consumption. Beer producers have expanded their product portfolio by launching NABs, however, they still experience different challenges in producing NABs with similar physicochemical and sensory characteristics to regular beers. The results of the present study on physicochemical quality characteristics of NABs, such as pH, bitterness, colour, TSS, and vitamin B_2_ content revealed that NABs, in general, are similar to alcoholic beers. This assortment can be also classified as a good source of minerals and antioxidant phenolic compounds being a healthier alternative to regular beers due to the lack of alcohol-related harm.

Based on the research conducted with mostly young NAB consumers (below 35 years old), it was found that these highly educated and high-income consumers mostly drink flavoured NABs followed by light NABs. They associated NAB with refreshment/thirst, driving, relaxation, a fruit beverage, and sobriety. They appreciated NAB for being able to enjoy the taste and aroma of beer without giving up daily habits such as driving or physical activity. The main factors important for the surveyed consumers were taste, availability in stores, price, brand, and friends’ opinions. No significant differences were noted between the indications of men and women for all factors determining the purchase of NAB except for the presence of sugar. This factor was more crucial for women than men.

The results of this study showed that young consumers are interested in consuming NAB. The advertising campaigns for these products should be aimed at communicating that NABs are not inferior to their alcoholic counterparts in terms of the selected nutritional and health-promoting compounds. It could also convince beer consumers to shift from the consumption of traditional beer to NAB and contribute to lower alcohol consumption, which is in line with the WHO public health priority of reducing the use of alcohol.

## Materials and methods

A total of 15 different NABs (12 different brands) were bought in Polish grocery shops in mid-2022: five brand lager beers, five craft IPA and five wheat beers. Before laboratory analyses, all beers were degassed using an ultrasonic bath until each sample was completely degassed. For determination of colour, TPC, and vitamin B_2_, beer samples were centrifuged at 12,000 *g* for 5 min (MiniSpin plus centrifuge, Eppendorf, Hamburg, Germany).

### Determination of pH, total soluble solids, colour and bitterness

The pH measurements were carried out with a pH meter (SevenCompact, Mettler Toledo GmbH, Germany) equipped with a glass electrode.

TSS content, expressed in ^o^Brix value, was determined using a hand-held refractometer (PAL Bx/RI, Atago Co., Ltd., Japan).

Colour of NAB was determined spectrophotometrically according to the method described in PN-A-79093-5:2000^58^ and expressed in EBC units. The absorbance of each beer was measured against demineralized water at λ = 430 nm. Beer colour in EBC units was calculated as: EBC = 25 x *A* x *f*, where *A* was the absorbance and *f* was the dilution factor.

Bitterness of NAB was determined spectrophotometrically according to the method described in PN-A-79093-12:2000^59^. Briefly, 2 mL of degassed beer was placed into a 10 mL screw-top tube, 0.05 mL of 6 M hydrochloric acid and 2 mL of isooctane were added. The flask was shaken for 5 min using a vortex and the emulsion was allowed to settle (if required). The absorbance of the isooctane extract was measured against pure isooctane in a 10 mm quartz microcuvette at λ = 275 nm. Bitterness was calculated as absorbance (*A*) x 50 and expressed in the International Bitterness Unit (IBU).

All determinations were performed in triplicate.

### Determination of total phenolic content

Total phenolic content (TPC) was determined using the Folin-Ciocalteu reagent according to the method of Singleton and Rossi^60^ with its adaptation to 48-well microplates as described by Swat et al.^61^. Briefly, 0.01 mL of degassed beer was mixed with 0.05 mL of Folin-Ciocalteu reagent. After 3 min, 0.15 mL of 20% Na_2_CO_3_ was added, followed by 0.79 mL of demineralized water. After 2 h in the dark at room temperature, the absorbance at λ = 765 nm was measured using a microplate spectrophotometer. The TPC was expressed as mg of gallic acid equivalents (mg GAE/L). The analysis was performed in triplicate.

### Determination of vitamin B_2_

Analysis of vitamin B_2_ content in beer samples was performed at room temperature using a Waters Alliance e2695 high-performance liquid chromatograph equipped with a Symmetry C18 column (150 mm × 3.9 mm, 5 μm) fitted with a µBondapak C18 cartridge guard column (Waters, Millford, MA, USA). A mobile phase gradient was applied: methanol (solvent A) and 0.05 M ammonium acetate pH 6.0 (solvent B) according to the following mobile phase gradient: a linear increase from 25 to 30% A in 5 min, a linear increment to 100% A during next 1 min and a return to initial conditions over the next 9 min. The flow rate was 1 mL/min. The injection volume was 0.020 mL. Eluate was detected using a Waters 996 photodiode-array detector set at λ = 450 nm and a Waters 2475 fluorescence detector set at 450/530 nm as excitation and emission wavelengths. Vitamin B_2_ forms (FAD, FMN and riboflavin) were identified by comparing the retention times and absorption spectra with their standards. Each sample was analysed in triplicate. Vitamin B_2_ quantification was performed using the external standard method with riboflavin as a standard^62,63^.

### Determination of minerals

The concentrations of selected macroelements – Ca, K, Mg and Na were determined using microwave plasma-atomic emission spectrometry (MP-AES 4210, Agilent Technologies, Melbourne, Australia) as described by Pires et al.^64^. Briefly, the macroelements were determined after sample dilution with 1 M nitric acid at 1:4 ratio (sample: nitric acid). The spectroscopic measurements were performed for Ca (λ = 417.2 nm), Mg (λ = 518.4 nm), K (λ = 769.9 nm) and Na (λ = 589.6 nm and 330.2 nm). Two seven-point calibration curves were prepared using standards at the levels adjusted to the expected concentration of appropriate mineral in analysed samples.

The content of P in the beer was determined spectrophotometrically as described by Gliszczyńska-Świgło and Rybicka^65^. The formation of molybdophosphoric acid from orthophosphate and molybdate under acidic conditions followed by reduction to molybdenum blue was conducted in 48-microwell plates. The absorbance measured at λ = 823 nm was proportional to the amount of P in beer. Quantification of P was performed using an external standard method with P for AAS as a standard.

All analyses were performed in triplicate.

### Nutritional requirements for vitamin B_2_ and minerals

The daily requirements for vitamin B_2_ and minerals were established at the level of nutrient reference values (NRVs) for riboflavin (1.4 mg), magnesium (375 mg), calcium (800 mg), potassium (2000 mg), and adequate intake (AI) for Na (2000 mg)^66,67^.

### Consumer study

The survey was related to the perception and consumption of NABs. It was conducted in accordance with the relevant guidelines and regulations. Participants were informed about the purpose of the survey and their participation was entirely voluntary. They were also informed that they would participate in the survey using their personal device, and that all data will be de-identified and reported only in the aggregate. The survey ensured anonymity. Each participant gave informed consent by confirming the terms and conditions before taking part in the survey. Due to the anonymity of the survey and the collection of non-sensitive information, the approval of the institutional Committee on Research Ethics was not required^68^. The data were collected through an online questionnaire created with Google Forms and distributed using peer-to-peer contacts and social media (Facebook) channels in May and June 2022. A total of 209 respondents took part in the survey. The questionnaire (Supplementary Material) was structured into two sections: socio-demographic (questions related to gender, age, place of residence, and monthly income; Table 3) and NAB consumption, and purchasing habits. The latter one included the following questions: (i) Have you ever consumed NAB?, (ii) How often do you consume NAB?, (iii) What type of NAB do you prefer?, (iv) Why do you drink NAB?, and (v) Which sensory attributes and factors influence the NAB purchasing decisions? Additionally, participants were asked to name the words they associate with NAB.

### Statistical analysis

Statistical analyses were carried out using Statistica 13.3 (2017) (Stat-Soft, Inc., Tulsa, OK, USA). The chi-square test was used to determine whether there is a significant association between two qualitative variables (gender vs. factor influencing purchase decisions). The V-Cramer coefficient was calculated to determine the strength of the association between the variables. Prior to statistical analysis, the original 5-point Likert scale was aggregated to a 3-point scale. Responses categorized as ‘not important at all’ and ‘rather unimportant’ were combined into the ‘not important’ group, while ‘rather important’ and ‘very important’ were combined into the ‘important’ category. The response ‘difficult to say’ was retained as a separate group. This transformation has enabled a proper statistical analysis of the results obtained and their clearer graphical presentation (Fig. 3B).

The physicochemical data are presented as mean ± SD for each product. All data were submitted to one-way analysis of variance (ANOVA). The significance of differences between mean values was determined by the least significant differences test (LSD) at α = 0.05.

Principal Component Analysis (PCA) of mineral content in non-alcoholic and regular beers was carried out using the Unscrambler 7.0 software (CAMO, Oslo, Norway). Leave-one-out cross-validation was applied.

## Electronic supplementary material

Below is the link to the electronic supplementary material.

Supplementary Material 1

## Data Availability

The data supporting the results of this study are available from the corresponding author upon a reasonable request.

## Abbreviations

ABV: Alcohol by volume
FAD: Flavin adenine dinucleotide
FMN: Flavin mononucleotide
GAE: Gallic acid equivalent
IBU: International Bitterness Units
NAB: Non–alcoholic beer
PCA: Principal component analysis
TPC: Total phenolic content
TSS: Total soluble solids

## References

[1] WHO. Alcohol action plan 2022–2030. Geneva, Licence: CC BY-NC-SA 3.0 IGO. (2024).

[2] European Commission. Communication from the Commission to the Council, the European Parliament, the European Economic and Social Committee and the Committee of the Regions an EU strategy to support Member States in reducing alcohol related harm {Sect. (2006) 1358} {Sect. (2006) 1360} {Sect. (2006) 1411} /* COM/2006/0625 final. (2006). Retrieved from https://eur-lex.europa.eu/legal-content/EN/TXT/?uri=celex%3A52006DC0625. Accessed 4.01.2024.

[3] United Nations. The 2030 agenda for sustainable development. (2015). Retrieved from https://sdgs.un.org/goals. Accessed 27.10.2023.

[4] WHO. Global strategy to reduce the harmful use of alcohol. (2010).

[5] Cerveceros Latinoamericanos. Our commitment to responsible beer consumption. (2018). Retrieved from https://cerveceroslatinoamericanos.com/wp-content/uploads/2018/06/our-commitment.pdf. Accessed 11.10.2023.

[6] Heineken. (2024). Retrieved from https://www.theheinekencompany.com/sustainability-and-responsibility/responsible. Accessed 18.05.2024.

[7] OECD. Alcohol consumption (indicator). Retrieved from http//doi.10.1787/e6895909-en. Accessed 21.01.2024. (2024).

[8] Hendriks, H. F. J. Alcohol and human health: what is the evidence? *Ann. Rev. Food Sci. Technol.* **11**, 1–21. 10.1146/annurev-food-032519-051827 (2020). 32209032 10.1146/annurev-food-032519-051827

[9] Lee, D. Y. et al. Association between alcohol consumption pattern and the incidence risk of type 2 diabetes in Korean men: a 12-years follow-up study. *Sci. Rep.* **7**, 7322. 10.1038/s41598-017-07549-2 (2017). 28779170 10.1038/s41598-017-07549-2 PMC5544746

[10] What’s a Standard Drink Measurement?. *Rethinking Drinking*. National Institute of Alcohol Abuse and Alcoholism. Accessed 15 January 2025.

[11] Carlsberg Retrieved from https://www.carlsbergmarstons.co.uk/sustainability/our-ambitions/zero-irresponsible-drinking/. Accessed 18.05.2024.

[12] Salantă, L. C. et al. Non-alcoholic and craft beer production and challenges. *Processes* **8**, 1382. 10.3390/pr8111382 (2020).

[13] Sohrabvandi, S., Mousavi, S. M., Razavi, S. H., Mortazavian, A. M. & Rezaei, K. Alcohol-free beer: methods of production, sensorial defects, and healthful effects. *Food Rev. Int.* **26**, 335–352. 10.1080/87559129.2010.496022 (2010).

[14] Company, K. H. Limited. Global beer consumption by country in 2022. Retrieved from https://www.kirinholdings.com/en/newsroom/release/2023/1222_04.html. Accessed 25.05.2024.

[15] Waehning, N. & Wells, V. K. Product, individual and environmental factors impacting the consumption of no and low alcoholic drinks: a systematic review and future research agenda. *Food Qual. Prefer*. **117**, 105163. 10.1016/j.foodqual.2024.105163 (2024).

[16] Katainen, A. et al. Who buys non-alcoholic beer in Finland? Sociodemographic characteristics and associations with regular beer purchases. *Int. J. Drug Policy*. **113**, 103962. 10.1016/j.drugpo.2023.103962 (2023). 36746032 10.1016/j.drugpo.2023.103962

[17] Euromonitor International. Retrieved from https://www-1portal-1euromonitor-1com-10000526p0059.han3.ue.poznan.pl/analysis/tab Accessed 4.01.2024.

[18] Statista Revenue of non-alcoholic beer worldwide from 2014 to 2027. Retrieved from https://www.statista.com/forecasts/1091389/non-alcoholic-beer-market-size-worldwide. Accessed 22.12.2023.

[19] Nardini, M. An overview of bioactive phenolic molecules and antioxidant properties of beer: emerging Trends. *Molecules* **28**, 3221. 10.3390/molecules28073221 (2023). 37049984 10.3390/molecules28073221 PMC10096009

[20] Zambrzycka-Szelewa, E., Nalewajko-Sieliwoniuk, E., Zaremba, M., Bajguz, A. & Godlewska-Żyłkiewicz, B. The mineral profile of Polish beers by fast sequential multielement HR CS FAAS analysis and its correlation with total phenolic content and antioxidant activity by chemometric methods. *Molecules* **25**, 3402. 10.3390/molecules25153402 (2020). 32727164 10.3390/molecules25153402 PMC7436273

[21] Anderson, P., O’Donnell, A., Kokole, D., Llopis, E. J. & Kaner, E. data 2015–2020. *Int. J. Environ. Res. Public. Health*. **18**, 10347. 10.3390/ijerph181910347 (2021). Is buying and drinking zero and low alcohol beer a higher socio-economic phenomenon? Analysis of British survey data, 2015–2018 and household purchase. 10.3390/ijerph181910347 PMC8508356 34639647

[22] Worldcrunch How Gen Z is breaking Europe’s eternal alcohol habit. Retrieved from https://worldcrunch.com/culture-society/gen-z-alcohol-consumption. Accessed 20.05.2024).

[23] Muller, C., Neves, L. E., Gomes, L., Guimarães, M. & Ghesti, G. Processes for alcohol-free beer production: a review. *Food Sci. Technol.* **40**, 273–281 (2020).

[24] Beer Judge Certification Program. Style Guidelines. Beer Style Guidelines. (2021). Retrieved from http://www.bjcp.org. Accessed 25.01.2024.

[25] Ramsey, I., Yang, Q., Fisk, I. & Ford, R. Understanding the sensory and physicochemical differences between commercially produced non-alcoholic lagers, and their influence on consumer liking. *Food Chem. X*. **9**, 100114. 10.1016/j.fochx.2021.100114 (2021). 33532724 10.1016/j.fochx.2021.100114 PMC7822955

[26] Lafontaine, S. et al. Evaluating the chemical components and flavor characteristics responsible for triggering the perception of beer flavor in non-alcoholic beer. *Foods* **9**, 1914. 10.3390/foods9121914 (2020). 33371467 10.3390/foods9121914 PMC7767514

[27] Santos, D. C. et al. Commercial craft beers of midwest Brazil: biochemical and physicochemical properties and their relationship with its sensory profile. *Food Sci. Technol.* **43**, e112222. 10.1590/fst.112222 (2023).

[28] Blackmore, H., Hidrio, C. & Yeomans., M. R. How sensory and hedonic expectations shape perceived properties of regular and non-alcoholic beer. *Food Qual. Pref*. **99**, 104562. 10.1016/j.foodqual.2022.104562 (2022).

[29] Lafontaine, S. et al. Characterizing volatile and nonvolatile factors influencing flavour and American consumer preference toward nonalcoholic beer. *ACS Omega*. **5** (36), 23308–23321. https://doi.org/10.1021%2Facsomega.0c03168 (2020). 32954182 10.1021/acsomega.0c03168 PMC7495743

[30] Ramsey, I. et al. Using a combined temporal approach to evaluate the influence of ethanol concentration on liking and sensory attributes of lager beer. *Food Qual. Pref*. **68**, 292–303. 10.1016/j.foodqual.2018.03.019 (2018).

[31] Ivanova, N. et al. The impact of varying key sensory attributes on consumer perception of beer body. *Food Qual. Pref*. **112**, 105004. 10.1016/j.foodqual.2023.105004 (2023).

[32] Sikorska, E. et al. Simultaneous analysis of riboflavin and aromatic amino acids in beer using fluorescence and multivariate calibration methods. *Anal. Chim. Acta*. **613**, 207–217 (2008). 18395060 10.1016/j.aca.2008.02.063

[33] USDA. Retrieved from https://fdc.nal.usda.gov/fdc-app.html#/. Accessed 27.10.2023.

[34] Sakuma, S., Rikimaru, Y., Kobayashi, K. & Kowaka, M. Sunstruck flavor formation in beer. *Am. Soc. Brew. Chem.* **49**, 162–165 (1991).

[35] Saura-Calixto, F., Serrano, J. & Perez-Jimenez, J. What contribution is beer to the intake of antioxidants in the diet? In Beer in Health and Disease Prevention (ed Preedy, V. R.) 441–456 (Department of Nutrition and Dietetics King’s College, (2009).

[36] Vanderhaegen, B., Neven, H., Verachtert, H. & Derdelinck, G. The chemistry of beer aging – a critical review. *Food Chem.* **95**, 357–381 (2006).

[37] Habschied, K., Košir, I. J., Krstanović, V., Kumrić, G. & Mastanjević, K. Beer polyphenols – bitterness, astringency, and off-flavors. *Beverages* **7**, 38. 10.3390/beverages7020038 (2021).

[38] Gliszczyńska-Świgło, A., Ignaszewska, A. & Tyrakowska, B. Quality of selected Polish beers evaluated on the basis of the polyphenol content and the antioxidant capacity. In *Wybrane problemy oceny jakości żywności*, [eds. Żuchowski, J. & Zieliński, R.) 43–51 (Wydawnictwo Politechnika Radomska, 2010).

[39] Mitić, S. S. et al. Phenolic profiles and total antioxidant capacity of marketed beers in Serbia. *Int. J. Food Prop.* **17**, 908–922. 10.1080/10942912.2012.680223 (2014).

[40] Nardini, M. & Foddai, M. S. Phenolics profile and antioxidant activity of special beers. *Molecules* **5**, 2466. 10.3390/molecules25112466 (2020). 10.3390/molecules25112466 PMC7321254 32466403

[41] Carvalho, D. O. & Guido, L. F. A review on the fate of phenolic compounds during malting and brewing: Technological strategies and beer styles. *Food Chem.* **372**, 131093. 10.1016/j.foodchem.2021.131093 (2022). 34619521 10.1016/j.foodchem.2021.131093

[42] Salantă, L. C. et al. Functionality of Special Beer processes and potential health benefits. *Processes* **8** (12), 1613. 10.3390/pr8121613 (2020).

[43] Borșa, A. et al. Effects of Botanical Ingredients Addition on the Bioactive Compounds and Quality of Non-Alcoholic and Craft Beer. *Plants*, 11(15), (1958). 10.3390/plants11151958 (2022). 10.3390/plants11151958 PMC9370188 35956436

[44] Alcázar, A., Pablos, F., Martín, M. A. & González, A. G. Multivariate characterisation of beers according to their mineral content. *Talanta* **57**, 45–52. 10.1016/s0039-9140(01)00670-1 (2022). 10.1016/s0039-9140(01)00670-1 18968603

[45] Bamforth, C. W. Nutritional aspects of beer—a review. *Nutr. Res.* **22**, 227–237 (2002).

[46] Gajek, M., Wysocki, P., Pawlaczyk, A. & Sać, Ł. Szynkowska-Jóźwik, M. I. The elemental profile of beer available on Polish market: analysis of the potential impact of type of packaging material and risk assessment of consumption. *Molecules* **27**, 2962. 10.3390/molecules27092962 (2022). 35566304 10.3390/molecules27092962 PMC9100925

[47] Rajkowska, M., Holak, M. & Protasowicki, M. Makro- i mikroelementy w wybranych asortymentach piwa (macro- and microelements in some selected assortments of beer). *Żywność Nauka Technologia Jakość (Food Sci. Technol. Qual)*. **2**, 112–118 (2009).

[48] Voica, C., Magdas, D. A. & Feher, I. Metal content and stable isotope determination in some commercial beers from Romanian markets. *J. Chem. 2015 Article ID*. **192032**, 10. 10.1155/2015/192032 (2015).

[49] Statista Consumer Insights Global as of the Statista Consumer insights Global survey. Target audience: Non-alcoholic beer drinkers in the UK. Retrieved from https://www-1statista-1com-1s8fui2s40018.han3.ue.poznan.pl/study/144762/target-audience-non-alcoholic-beer-drinkers-in-the-united-kingdom/. Accessed 27.10.2023.

[50] Statista Consumer Insights Global as of the Statista Consumer insights Global survey. Target audience: Non-alcoholic beer drinkers in the U.S. Retrieved from https://www-1statista-1com-1s8fui2s40018.han3.ue.poznan.pl/study/137175/target-audience-non-alcoholic-beer-drinkers-in-the-united-states/. Accessed 27.10.2023.

[51] Statista Consumer Insights Global as of the Statista Consumer insights Global survey. Target audience: Non-alcoholic beer drinkers in Germany. Retrieved from https://www-1statista-1com-1s8fui2s40018.han3.ue.poznan.pl/study/137170/target-audience-non-alcoholic-beer-drinkers-in-germany/. Accessed 27.10.2023.

[52] Kompania Piwowarska. 0% Alkoholu 100% smaku. Raport Kompanii Piwowarskiej o piwach bezalkoholowych w 2022 roku. ((% Alcohol. 100 Taste. Kompania Piwowarska’s report on non-alcoholic beers in 2022). Retrieved from http://www.kp.pl/files/cms/1606388039_0_Alkoholu_100_Procent_Smaku_Raport_Kompanii_Piwowarskiej_o_piwach_bezalkoholowych_w_2020_roku.pdf. Accessed 29.10.2023.

[53] Brewbound Retrieved from https://www.brewbound.com/news/heineken-usa-releases-non-alcoholic-beer-in-us/. Accessed 29.10.2023.

[54] Da Jardim, C. Sensory profile, consumer preference and chemical composition of craft beers from Brazil. *Beverages* **4**, 106. 10.3390/beverages4040106 (2018).

[55] SW Research. Retrieved from https://swresearch.pl/news/piwo-bezalkoholowe-juz-nie-tylko-dla-kierowcow. Accessed 27.10.2023.

[56] Staub, C., Contiero, R., Bosshart, N. & Siegrist, M. You are what you drink: stereotypes about consumers of alcoholic and non-alcoholic beer. *Food Qual. Prefer*. **101**, 104633. 10.1016/j.foodqual.2022.104633 (2022).

[57] Silva, A. P. et al. What’s in a name? The effect of congruent and incongruent product names on liking and emotions when consuming beer or non-alcoholic beer in a bar. *Food Qual. Prefer*. **55**, 58–66. 10.1016/j.foodqual.2016.08.008 (2017).

[58] PN-A-79093-5:2000. Piwo - Metody badań - Oznaczanie barwy (Beer - Test methods - Determination of colour).

[59] PN-A-79093-12. Piwo - Metody badań - Oznaczanie wartości goryczy metodą spektrofotometryczną (Beer - Test methods - Determination of bitterness values by spectrophotometric method). (2000).

[60] Singleton, V. L. & Rossi, J. A. Colorimetry of total phenolics with phosphomolybdic-phosphotungstic acid reagents. *Am. J. Enol. Vitic*. **16**, 144–158 (1965).

[61] Swat, M., Rybicka, I. & Gliszczyńska-Świgło, A. Characterization of fulvic acid beverages by mineral profile and antioxidant capacity. *Foods* **8**, 605. 10.3390/foods8120605 (2019). 31766604 10.3390/foods8120605 PMC6963745

[62] Gliszczyńska-Świgło, A. & Koziołowa, A. Chromatographic determination of riboflavin and its derivatives in food. *J. Chromatogr. A*. **881**, 285–297. 10.1016/s0021-9673(00)00200-4 (2000). 10905712 10.1016/s0021-9673(00)00200-4

[63] Gliszczyńska-Świgło, A. & Rybicka, I. Minerals and vitamin B_2_ in flavoured dairy products. *J. Food Compos. Anal.* **124**, 105695. 10.1016/j.jfca.2023.105695 (2023).

[64] Pires, L. N., Dias, F. & Teixeira, L. S. G. Assessing the internal standardization of the direct multi-element determination in beer samples through microwave-induced plasma optical emission spectrometry. *Anal. Chim. Acta*. **1090**, 31–38. 10.1016/j.aca.2019.09.033 (2019). 31655643 10.1016/j.aca.2019.09.033

[65] Gliszczyńska-Świgło, A. & Rybicka, I. Fast and sensitive method for phosphorus determination in dairy products. *J. Consum. Prot. Food Saf.* **16**, 213–218. 10.1007/s00003-021-01329-x (2021).

[66] Regulation, E. U. & No 1169/2011 of the European Parliament and of the Council of 25 October. 2011 on the provision of food information to consumers, amending Regulations (EC) No 1924/2006 and (EC) No 1925/2006 of the European Parliament and of the Council, and repealing Commission Directive 87/250/EEC, Council Directive 90/496/EEC, Commission Directive 1999/10/EC, Directive 2000/13/EC of the European Parliament and of the Council, Commission Directives 2002/67/EC and 2008/5/EC and Commission Regulation (EC) No 608/2004.

[67] EFSA NDA Panel (European Food Safety Authority Panel on Nutrition, Novel Foods and Food Allergens) et al. Scientific opinion on the dietary reference values for sodium. *EFSA J.* **17**, 5778, 191 (2019). 10.2903/j.efsa.2019.5778 PMC7009309 32626425

[68] Committee on Research Ethics at the Poznań University of Economics and Business. The ethical recommendations for researchers, (2024). https://ue.poznan.pl/uniwersytet/badania-naukowe-na-uep/komisja-ds-etyki-badan-naukowych/

