# Supplementary material for: Quality characteristics and consumer perception of non-alcoholic beers in the context of responsible alcohol consumption
Source: Sci Rep. 2025 Feb 28;15:7145. doi: 10.1038/s41598-025-89833-0 (PMC11871341; doi:10.1038/s41598-025-89833-0)
Supplement: Supplementary file 1 — Supplementary Material 1 [file 41598_2025_89833_MOESM1_ESM.docx]

**S1. Questionnaire on the consumer attitudes towards non-alcoholic beer**

Dear Sir/Madam,

We kindly ask for your support in collecting data for a study on consumer attitudes towards non-alcoholic beer. The survey is completely anonymous and the answers provided will be used solely for research purposes. We invite you to complete the questionnaire and encourage you to share it with your friends who might also be interested in participating. Completing the questionnaire will take approximately 5 minutes. Thank you very much for your time and cooperation.

* Required Questions

**1. Have you ever consumed non-alcoholic beer?** *
*Mark only one answer.

Yes

No (Skip to demographics)

**2. How often do you consume non-alcoholic beer?***

*Mark only one answer.

Every day

Several times a week

Once a week

Several times a month

Several times a year

**3. What type of non-alcoholic beer do you prefer?** *
*Mark only one answer.

Light

Dark

Wheat

Flavoured

Other …….

**4. Why do you drink non-alcoholic beer?** *
*You may select multiple answers.

For refreshment

I cannot consume alcohol for health reasons

I am on a diet

The ability to drive after drinking

For taste reasons

For the need to relax

**5. Please provide one word that comes to mind when you hear "non-alcoholic beer”:**

………………………………………………………………………………………………………………………………………………………………………..

**6. Please evaluate the importance of the following factors when purchasing non-alcoholic beer*.**
**Choose on a scale between “Not important at all” and “Very important” regarding the following factors.**

*Mark only one answer per row. Mark your answer with X.

| **Factors** | **Not important at all** | **Rather unimportant** | **Difficult to say** | **Rather important** | **Very important** |
| --- | --- | --- | --- | --- | --- |
| **Taste** |  |  |  |  |  |
| **Presence of sugar** |  |  |  |  |  |
| **Colour** |  |  |  |  |  |
| **Beer style** |  |  |  |  |  |
| **Energy value** |  |  |  |  |  |
| **Availability** |  |  |  |  |  |
| **Habit** |  |  |  |  |  |
| **Price** |  |  |  |  |  |
| **Brand** |  |  |  |  |  |
| **Label** |  |  |  |  |  |
| **Country of origin** |  |  |  |  |  |
| **Internet opinions** |  |  |  |  |  |
| **Opinions of friends** |  |  |  |  |  |

**Demographic Questions**

**Gender** *
*Mark only one answer.

Female

Male

Other

Prefer not to say

**Age** *
*Mark only one answer.

Below 18 years

18-25 years

26-35 years

36-45 years

46-55 years

Over 55 years

**Place of Residence** *
*Mark only one answer.

Village

City below 50,000 inhabitants

City 50,000-150,000 inhabitants

City 150,000–500,000 inhabitants

City over 500,000 inhabitants

**Education** *
*Mark only one answer.

Primary

Vocational

Secondary

Higher (engineer/bachelor)

Higher (master’s degree)

**Socio-Occupational Status** *
*Mark only one answer.

Student (high school)

Student (first degree)

Student (second degree)

Unemployed

Housekeeping

White-collar worker

Blue-collar worker

Retired

Other

**Monthly Income** *
*Mark only one answer.

No income

Not sufficient

Can afford only basic or selected things

Can afford anything

Can afford anything and save
